# Outcome of older adults at risk of frailty

**DOI:** 10.1002/agm2.12181

**Published:** 2021-11-12

**Authors:** Venugopalan Gunasekaran, Manicka Saravanan Subramanian, Vishwajeet Singh, Aparajit Ballav Dey

**Affiliations:** ^1^ Department of Geriatric Medicine All India Institute of Medical Sciences New Delhi India; ^2^ Department of Geriatric Medicine Jawaharlal Institute of Postgraduate Medical Education and Research Puducherry India

**Keywords:** At risk of frailty, frailty syndrome, older adults, prospective

## Abstract

**Background:**

The integral part of the definition of frailty is the outcome associated with it. Older adults at risk of frailty are in the process of becoming frail. This study looked at the clinical characteristics and outcomes of older adults at risk of frailty.

**Methodology:**

The study population was selected from outpatient department of the geriatric medicine department in a tertiary care hospital. Older adults identified as at risk of frailty were assessed at baseline and then followed up after 1 year for the composite primary outcome of death, falls, hospitalization, and self‐rated poor quality of life in the follow‐up period.

**Results:**

The study included 324 older adults who had completed 1‐year follow up. Mean (SD) age was 74.49 (4.58) years, and males were 241 (74.15%). Frail and pre‐frail at baseline among the study population were 31.17% and 61.11%, respectively. The primary outcome occurred in 43 (13.27%) patients. Poor baseline IADL was significantly associated with primary outcome at the end of 1 year.

**Conclusion:**

An unfavorable outcome in older adults at risk of frailty was significantly higher and independent of their baseline frailty status. Poor baseline IADL value may be considered as a predictor for primary outcome at 1 year of follow up.

## Graphical abstract

The integral part of the definition of frailty is the outcome associated with it. Older adults at risk of frailty are in the process of becoming frail. This study looked at the clinical characteristics and outcomes of older adults at risk of frailty. The study population was selected from outpatient department of the geriatric medicine department in a tertiary care hospital. Older adults identified as at risk of frailty were assessed at baseline and then followed up after 1 year for the composite primary outcome of death, falls, hospitalization, and self‐rated poor quality of life in the follow‐up period. The study included 324 older adults who had completed 1 year of follow up. Mean (SD) age was 74.49 (4.58) years, and males were 241 (74.15%). Frail and pre‐frail at baseline among the study population were 31.17% and 61.11%, respectively. The primary outcome occurred in 43 (13.27%) patients. Poor baseline IADL was significantly associated with primary outcome at the end of 1 year. An unfavorable outcome in older adults at risk of frailty was significantly higher and independent of their baseline frailty status. Poor baseline IADL value may be considered as a predictor for primary outcome at 1 year of follow up.

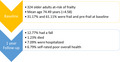

## 1 INTRODUCTION

A consensus definition describes frailty as “a medical syndrome with multiple causes and contributors that is characterized by diminished strength, endurance, and reduced physiologic function that increases an individual's vulnerability for developing increased dependency and/or death.”^1^ When the phenotype model was described, the outcomes used to define frailty were falls, disability, hospitalization, and mortality.^2^ The syndrome or state that predisposes older adults to these outcomes is central to the theme of frailty. The phenotype model had five variables, which predicted these outcomes when three or more were present. The cumulative deficit model used many variables to predict these outcomes; the 36 variable questionnaire predicted these outcomes when nine or more variables were present (Frailty Index 0.25 or more).^3^ The variables used to identify frailty can change with the scales or model used. But the goal is to find the group of older adults, labelled as frail, who are at higher risk of these outcomes when compared to those who are robust.

The screening of frailty in older adults is not mandatory for all, particularly for those below age 70 years. Asia‐Pacific guidelines recommend that older adults 70 years of age and above or those who had unintentional weight loss of >5% of body weight in the past year be screened routinely for frailty in a clinical setting.^4^ The screening criteria can vary with the societies' recommendation, but generally, all older adults 70 years and above may benefit from frailty screening.

Western models used for assessment of frailty in the Indian population may not reflect the actual status. The objective of our study was to screen older adults who are at risk of frailty and follow them prospectively for the outcome of interest. Based on these primary outcomes, we intended to develop a suitable model for the Indian population for identification of frailty.

## 2 METHODOLOGY

The study was conducted in Geriatric Medicine Outpatient Department (OPD) at the All India Institute of Medical Sciences, New Delhi, from 2017 to 2020. The study was a prospective observational cohort study of older adults 70 years and above who attended Geriatric Medicine OPD were screened for the risk of frailty and inclusion in the frailty registry. The risk of frailty was defined by loss of weight or self‐reported decline in physical activity or one or more falls in the past six months. So, older adults 70 years and above with any of the three risk factors were included after written informed consent. Older adults with severe depression, severe cognitive impairment (Hindi Mental State Examination score <18), severe osteoarthritis knee, severe heart failure, decompensated liver or kidney disease, or severe anaemia were excluded from the study.

Detailed baseline data were collected during study recruitment, including demographic data, anthropometry, medical history, physical function, functionality, and mental status. Health‐related conditions and medications were identified by reviewing health records and blister packs. Socioeconomic status was assessed by a periodically updated standard Indian scale.^5^ A comprehensive geriatric assessment was carried out to find any geriatric syndromes, vision by Snellen or E chart and hearing impairment by Whispering test.^6^ A fall was defined as an event that results in a person coming to rest inadvertently on the ground or floor or other lower level.^7^ Orthostatic hypotension measured using a digital sphygmomanometer (Omron^TM^ 7310) uses an oscillometric method.^8^ Polypharmacy was defined as taking ≥5 medications.^9^ Functional status assessed by Barthel Activity Dependent Daily Living (ADL), Lawton's Instrumental activity dependent daily living (IADL). Physical performance assessed by Timed up and Go (TUG) test, Short physical performance battery (SPPB), and Functional reach test (FRT).^10^, ^11^, ^12^, ^13^ Depression and nutritional status were assessed by Geriatric Depression Scale short form (GDS SF) and Mini nutritional assessment short form (MNA‐SF) respectively.^14^, ^15^ Cognition assessed by Hindi Mental status examination (HMSE),^16^ and frailty was defined by Fried's phenotype model.^2^ Chair raise test done with a standardized armless chair, and the time taken was measured by a stopwatch. The 1 kg arm lift was measured by the standard dumbbell. Gait speed was assessed by the time taken to walk 4 meters at the usual pace. Grip strength measured using a hand dynamometer (JAMAR; Sammons Preston, Rolyon) by Southampton protocol,^18^ value of the dominant hand was considered. Mid‐arm circumference (MAC) and mid‐thigh circumference were measured when the subject was sitting in a relaxed position with arm‐by‐side and in lying position, respectively. MAC was measured at the midpoint between the acromion and olecranon process, and the thigh circumference was measured between the midpoint between the inguinal crease and the proximal border of the patella (Appendix S1).

The telephonic follow up was done after completion of 1 year. For those who have not responded to the initial follow‐up call, two more calls were made at 1‐month interval each. Some participants were followed up when they come for scheduled outpatient visit. Participants who completed at least one follow‐up call or visit after 1 year of recruitment were included in the analysis. The older adults who had not completed follow up were excluded from the study.

The primary outcome was defined as a composite event of history of hospitalization in the 1‐year follow‐up period or history of fall in the 1‐year follow‐up period or self‐rated poor overall health status during follow‐up call/visit or death in the follow‐up period.

### 2.1 Statistical analysis

The data were maintained in an Excel spreadsheet and analyzed using STATA‐SE (Version 14.2) (StataCorp). Normality assumption tested using the Kolmogorov‐Smirnov test and, accordingly, quantitative data were reported as the mean (standard deviation (SD)). The qualitative variables of the study participant were reported as numbers with percentages. To find the association between the categorical variables, chi square test or Fisher exact test were used according to the frequency distribution, and for comparing quantitative measures between the two groups, t‐test was used. To find out the factors associated with the primary outcome, stepwise multivariable logistic regression analysis approach was used and the results were presented in the form of odds ratio (95% confidence interval). Multicollinearity and confounders were also explored. Variables that were found to be significant at the level of 25% under crude analysis and/or clinically relevant were considered for stepwise procedure with the entry probability 15% and removal probability 20%. A *p* value of less than 0.05 was considered as statistically significant.

## 3 RESULTS

Out of the 550 older adults assessed for eligibility, only 324 had completed at least one follow up and included in the analysis. The baseline characteristics of study participants are given in Table 1. The mean (SD) age of the study population was 74.49 (4.58) years. The male population was predominant (74.15%). The primary outcome occurred in 13.27% of the overall study population. Only 10 (3.08%) lived alone; the rest lived with either spouse or children or both. The detailed baseline and follow up information of the characteristics are presented in Table 1.

**TABLE 1:** Characteristics of the study population

Characteristics	N = 324
Age in years, Mean (SD)	74.49 (4.58)
Gender, N (%)	
Male	241 (74.15%)
Female	84 (25.85%)
H/o falls	59 (18.15%)
Decline in physical activity	211 (64.92%)
H/o weight loss	91 (28.00%)
Difficulty in balance	111 (34.15%)
Change in gait	122 (37.54%)
Completed years of education, Mean (SD)	8.53 (5.74)
Marital status	
Widowed	103 (31.69%)
Married	222 (31.69%)
Socioeconomic class	
Upper class	66 (20.31%)
Upper middle	105 (32.31%)
Lower middle	83 (25.54%)
Upper lower	63 (19.38%)
Lower	8 (2.46%)
BMI in kg/m^2^, Mean (SD)	23.75 (4.58)
Underweight	43 (13.23%)
Normal	48 (14.77%)
Overweight	56 (17.23%)
Obese	178 (54.77%)
Waist hip ratio, Mean (SD)	1.39 (0.96)
Number of medications, Mean (SD)	4.77 (2.14)
Orthostatic hypotension	28 (8.64%)
Previous hospitalization	124 (38.15%)
Previous surgery	159 (48.92%)
3 kg weight loss in 3 months	8 (2.46%)
Primary Outcome at 1 year of follow up	43 (13.27%)
Hospitalization	23 (7.09%)
Fall	41 (12.77%)
Death	4 (1.23%)
Poor overall health	22 (6.79%)

The study population was categorized into two groups based on outcome as those with no outcome and primary outcome. In anthropometric measures, those without any outcome had significantly higher BMI (23.96 vs. 22.43 kg/m^2^), and more proportion of them were in the obese category (57.65% vs. 37.21%) than those with the primary outcome. Gait speed was slow, and the Timed‐Up‐Go test was high in those with the primary outcome (Table 2). The muscle mass measured by the upper arm and mid‐thigh were significantly lower in those with the primary outcome. It was also associated with a significantly lower mini‐nutritional assessment score. Both baseline Basic (BADL) and Instrumental (IADL) Activities of Daily living score was significantly lower in those with any negative outcome, and their functional reach was also significantly lower.

**TABLE 2:** Demographic characteristics of the population based on the outcome

Variables	No outcome (N = 281)	Primary outcome (N = 43)	*p*‐value
Age in years, Mean (SD)	74.35 (0.23)	75.60 (1.10)	0.177
Sex			
Male	210 (74.73%)	31 (72.09%)	0.547
Female	71 (25.27%)	12 (27.91%)	
H/o weight loss	77 (27.40%)	14 (32.56%)	0.544
H/o falls	51 (18.15%)	8 (18.60%)	0.996
Decline in physical activity	179 (63.70%)	32 (74.42%)	0.243
Pain in Numerical Rating Scale (0 = no pain; 10 = max pain), Mean (SD)	1.59 (0.13)	1.18 (0.32)	0.350
No of medications, Mean (SD)	4.73 (0.13)	5.00 (0.33)	0.374
BMI in kg/m^2^, Mean (SD)	23.96 (4.55)	22.43 (4.61)	0.018
Underweight	36 (12.81%)	7 (16.28%)	0.028
Normal	36 (12.81%)	12 (27.91%)	
Overweight	47 (16.73%)	8 (18.60%)	
Obese	162 (57.65%)	16 (37.21%)	
Waist hip ratio, Mean (SD)	1.05 (0.08)	0.95 (0.01)	0.636
Orthostatic hypotension	24 (8.54%)	4 (9.52%)	0.761
Gait speed in meter/second, Mean (SD)	0.70 (0.01)	0.63 (0.03)	0.056
TUG score in seconds, Mean (SD)	14.00 (0.23)	15.73 (0.79)	0.003
Circumference in centimeter, Mean (SD)			
Right mid‐arm (cm)	26.55 (0.20)	24.58 (0.67)	<0.001
Left mid‐arm (cm)	26.28 (0.20)	24.43 (0.67)	0.001
Right mid‐thigh (cm)	38.83 (0.31)	36.72 (1.09)	0.017
Left mid‐thigh (cm)	38.68 (0.31)	36.18 (0.99)	0.004
Poor grip strength	217 (77.50%)	33 (78.57%)	0.818
Time for 5 times chair stand (s), Mean (SD)	16.78 (0.37)	17.81 (0.90)	0.255
No. of chair stand in 30 seconds, Mean (SD)	9.37 (0.21)	9.56 (0.59)	0.867
SPPB			
Functional impairment	224 (79.72%)	40 (90.91%)	0.067
Functional reach (inches), Mean (SD)	11.92 (0.18)	10.53 (0.60)	0.010
BADL score, Mean (SD)	19.33 (0.07)	18.86 (0.25)	0.021
IADL score, Mean (SD)	6.78 (0.10)	5.72 (0.35)	<0.001
HMSE, Mean (SD)	27.07 (0.20)	26.02 (0.64)	0.041
GDS SF, Mean (SD)	4.13 (0.18)	4.86 (0.50)	0.112
MNA‐SF score, Mean (SD)	10.93 (0.12)	10.04 (0.34)	0.007
Frailty			
Non‐frail	21 (7.47%)	4 (9.30%)	0.803
Pre‐frail	174 (61.92%)	24 (55.81%)	
Frail	86 (30.60%)	15 (34.88%)	

In the overall study population, 31.17% were frail, and 61.11% were pre‐frail. Older adults who were designated as at risk of frailty and found to be robust were 7.7%. In the population with the primary outcome, 34.88% were frail and 55.81% were pre‐frail. In those without any of these outcomes in the follow‐up period, 30.60% were frail, and 174 were pre‐frail (Table 2).

Univariable analysis shows that low BADL score [0.81 (0.67–0.99)], low IADL score [0.79 (0.68–0.91)], low BMI [0.72 (0.55–0.95)], increased TUG score [1.09 (1.02–1.17)], low functional reach test score [0.88 (0.79–0.97)], and low MNA‐SF score [0.82 (0.71–0.95)] were significantly associated with the primary outcome (Table 3).

**TABLE 3:** Univariable analysis of variables for predicting the composite outcome in the study population

Variable	Primary outcome	Z score	*p*‐value
	Unadjusted Odds Ratio (95% Confidence Interval)		
Age	1.05 (0.99–1.11)	1.64	0.181
HMSE	0.93 (0.86–1.00)	−1.79	0.047
GDS SF	1.07 (0.97– 1.17)	1.39	0.115
Pain in NRS	0.91 (0.78–1.07)	−1.09	0.351
No of medications	1.05 (0.91–1.22)	0.75	0.373
BADL	0.81 (0.67–0.99)	−2.06	0.031
IADL	0.79 (0.68–0.91)	−3.24	0.001
BMI	0.72 (0.55–0.95)	−2.32	0.020
Waist hip ratio	0.01 (0.00–2.08)	−1.69	0.077
Time to walk 4 meter	1.10 (0.99−1.22)	1.81	0.045
TUG score	1.09 (1.02–1.17)	2.51	0.005
Time for 5 times chair rise	1.02 (0.97–1.08)	0.97	0.255
Time for 1 kg arm lift	1.03 (0.94–1.12)	0.68	0.379
30 second chair rise	1.01 (0.92–1.11)	0.32	0.867
SPPB score	0.89 (0.77–1.04)	−1.39	0.136
Functional reach	0.88 (0.79–0.97)	−2.52	0.012
MNA‐SF score	0.82 (0.71–0.95)	−2.52	0.009

The results of the multivariable analysis are presented in Table 4. Under multivariate analysis, only IADL with a OR (95% CI) of 0.84 (0.71–0.99) predicted the poorer outcome and had an inverse relationship.

**TABLE 4:** Multivariable analysis to identify the predictors associate with poor outcome

Variable	Adjusted Odds Ratio (95% Confidence Interval)	Z score	*p*‐value
MNA‐SF score	0.89 (0.75–1.05)	−1.36	0.173
Functional reach	0.90 (0.81–1.01)	−1.75	0.079
TUG score	1.08 (0.99–1.19)	1.72	0.085
IADL	0.84 (0.71–0.99)	−2.01	0.044
SPPB	1.17 (0.94−1.45)	1.43	0.153

## 4 DISCUSSION

Out of the 324 older adults who completed 1 year of follow‐up, the primary outcome occurred in 43 (13.27%). The 4‐year incidence of frailty was 7.2% in the phenotype study.^2^ The study outcome and measurement were different, but the rate of primary outcome is high in our study. Among the baseline characteristics, there was no difference in age, gender, living status, educational status, or socioeconomic status. There was no association with a history of weight loss, as it was one of the screening criteria for determining those at risk of frailty. However, mean BMI was lower in those who had the primary outcome.

Those with primary outcome had a significant difference in anthropometric measures and physical performance. They all had a significantly lower muscle bulk as measured by mid‐arm and mid‐thigh circumference. Along with reduced muscle bulk, the mean grip strength of the group was also poor, though not statistically significant. But when the grip strength was adjusted for age & gender, there was no difference between the two groups. The gait speed was significantly lower in those with the primary outcome. The gait speed as determined for their age and gender was also slow. The Timed‐up‐Go score was also significantly higher. These findings suggest slowness in gait is an important predictor of poor outcomes in older adults.

Functional Reach Test (FRT), Short Physical Performance Battery (SPPB), and TUG score measure physical performance and predict fall risk and physical frailty. The SPPB score is significantly lower in those with a negative outcome. The lower muscle bulk and poor SPPB score, both a marker of probable sarcopenia, were associated with the primary outcome. In a comparative study by Lim et al., SPPB correlates more with physical frailty, and poor performance in SPPB can predict the at‐risk of the frail population.^19^ SPPB is the composite measure of gait speed, lower limb strength, and balance, whereas FRT measures only dynamic balance.^20^ Those who had less reach in the functional reach test (FRT) had more falls, and its association with outcome was statistically significant also. As seen with slow gait speed and prolonged TUG score, slowness in gait along with probable sarcopenia and poor FRT dramatically increases the risk of falls. Falls occurred in 12.77% of the study population and were a major contributor to the primary outcome. A history of falls in the past year was one of the selection criteria for the study. Older adults with a history of fall are at higher risk of future fall.^21^ This could be a confounding factor, but we have to also look at how poor physical performance measures might be an effect modifier as it would have led to past and future falls in these study participants.

The BMI was normal‐high in the population, and no difference was observed between the groups in the grip strength. In this study, the muscle density and body composition were not examined, and the possibility of sarcopenic obesity cannot be ruled out. The prospective data from the InCHIANTI study showed that though the population had higher BMI, the frailer population had high‐fat mass, poor muscle density, and low gait speed, similar to our data. Sarcopenia is part of the vicious frailty cycle, but it would be difficult to determine whether sarcopenia preludes frailty, as the nosology is unknown. The grip strength is insignificant because it only measures the upper body or regional strength. But the gait speed and the composite physical performance measures examine overall strength and ability to perform daily activities.^22^

IADL impairment was one of the most common associations with frailty in the community‐dwelling older adults.^23^ Frailty is considered as a continuum, and there was a consensus that by improving comorbid conditions, it can be reversible.^24^ A prospective study by Zhang et al. identified the frailer population was at risk of disability and falls in the future.^25^ A similar association was also observed in the EPIDOS study in community‐dwelling older adults using the phenotype model^26^ and in the hospitalized patients by Liang et al. using the cumulative deficit model.^27^ The relation between frailty and disability is very close as each of them predisposes to the other. In our study, IADL impairment is strongly associated with primary outcomes irrespective of baseline frailty status. The study suggests that physical performance measures were poor, and IADL impairment can be used to predict primary outcomes in older adults.

There are few limitations in the study. Though we tried to look at the significant variables to construct a model to predict these outcomes, we had only one variable (IADL) with statistical significance. Many subjects did not complete at least one follow up and the attrition rate was very high, partially due to the COVID‐19‐related attrition of health services.

## 5 CONCLUSION

Older adults who were at risk of frailty had a high incidence of primary outcomes at 1 year of follow up. Poor physical performance measures like slow gait speed, increased TUG score, and lower SPPB score were also associated with the primary outcomes. The primary outcome was independent of the baseline frailty status but associated with their functionality (baseline IADL). Screening for IADL impairment in the at‐risk frailty population can be done at primary care level, and rehabilitation can be initiated to prevent falls and poor outcomes.

## CONFLICT OF INTEREST

None of the authors report any conflict of interest.

## AUTHOR CONTRIBUTIONS

VG, MSS, and ABD contributed in study concept and design, acquisition of data, andinterpretation of data. VS contributed in analysis of data and interpretation of data. All authors contributed in the preparation of manuscript and final approval.

## CONSENT FOR PUBLICATION

Not applicable.

## Supporting information

Supplementary Material
