# Supplementary material for: Outcome of older adults at risk of frailty
Source: Aging Med (Milton). 2021 Nov 12;4(4):266–71. doi: 10.1002/agm2.12181 (PMC8711216; doi:10.1002/agm2.12181)
Supplement: Supplementary file 1 — Supplementary Material [file AGM2-4-266-s001.docx]

**Supplementary Appendix**

Study procedure is given in Figure 1


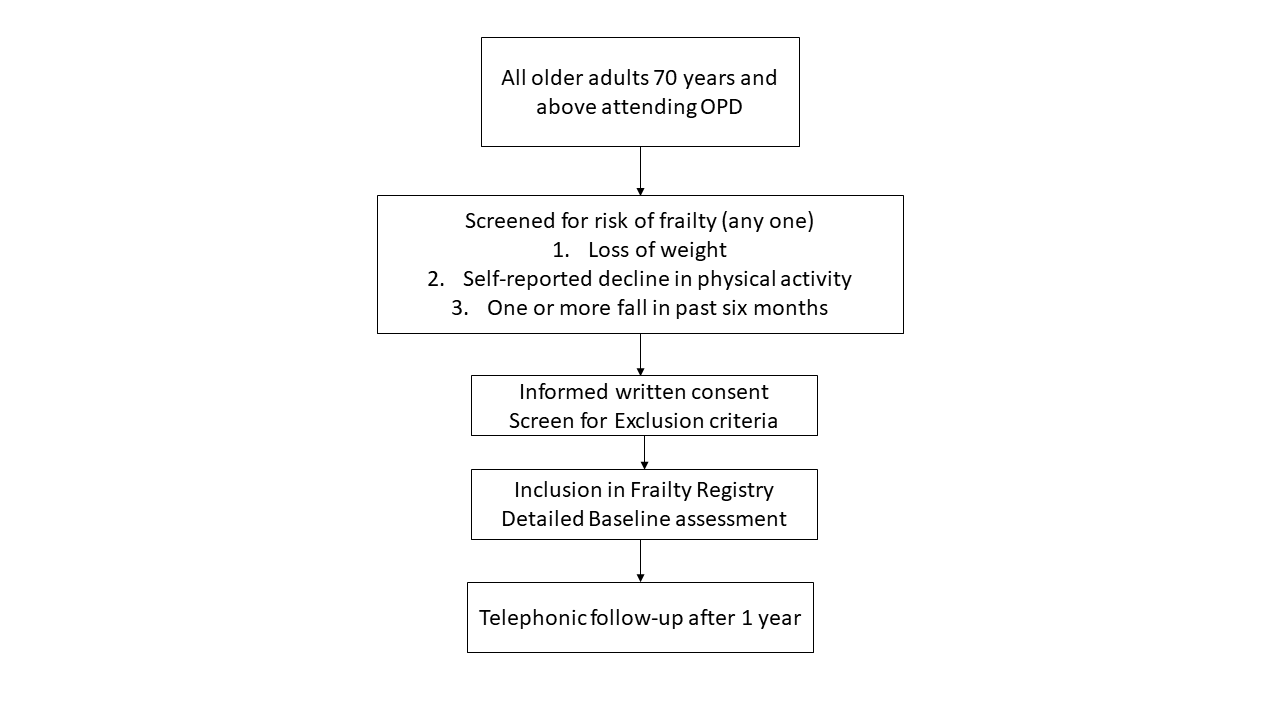


Figure 1: Study procedure

**Exclusion Criteria:**

- Severe depression (Geriatric Depression Scale-short form, GDS >10)
- Severe cognitive impairment (Hindi Mental Status Examination, HMSE score < 18)
- Parkinson disease
- Severe Osteoarthritis knee
- Severe Heart failure (NYHA class IV)
- Decompensated liver disease
- Chronic Kidney disease on dialysis
- Severe COPD
- Severe Anaemia (Hb < 8 gm/dl)
- Critically ill/Acute illness
- Participation in any other clinical trial

**Baseline Assessment:**

1. Socio-demographic details:
   1. Education
   2. Occupation
   3. Marital status
   4. Living status with family
   5. Socio-economic status: Modified Kuppuswamy scale (Table 1)
   6. Smoking history
   7. Alcohol history
   8. Physical activity
2. Questionnaire on health problems and current symptoms
3. Self-reported exhaustion
   1. Do you feel tired with your usual activities recently : Rarely / 1-2 (some) days a week / 3-4 (occasionally) days a week / 5-7 (most) days a week
   2. Do you feel that you do everything with effort: Rarely / 1-2 (some) days a week / 3-4 (occasionally) days a week / 5-7 (most) days a week
4. Name of current medications. Polypharmacy is defined as taking five or more medications..
5. Past medical history
6. Barthel Activities of Daily living
7. Lawton Instrumental Activities of Daily living
8. Life space assessment questionnaire
9. Mini Nutritional Assessment – short form
10. Quality of life questions
    1. How is your health: Excellent / Very good / Good / Fair / Poor
    2. Compared to one year ago, how would you rate your health in general now? : Much better / somewhat better / about the same / somewhat worse / much worse

**Clinical Assessment:**

1. Blood pressure - using a digital sphygmomanometer (Omron^TM^ 7310) uses an oscillometeric method. Orthostatic hypotension was measured by first measuring supine BP and standing BP at 1 minute and 3 minute. Fall of systolic BP more than 20 mm Hg or Diastolic BP more than 10 mm Hg at either 1 minute or 3 minute of standing is taken as orthostatic hypotension.
2. Antrhopometry
   1. Height
   2. Weight
   3. BMI
   4. Waist/Hip ratio
   5. Mid-arm circumference (both arms) – The participant sitting in relaxed position, arm-by-side, mid-point between the acromion and olecranon process
   6. Mid-thigh circumference (both thighs) – The participant in lying position, the thigh circumference measured between the mid-point between the inguinal crease and the proximal border of the patella
   7. Time to walk 4 meters – time noted for usual pace
   8. Gait speed – time taken to walk 4 meters at usual pace
   9. Grip strength – measured using a hand dynamometer (JAMAR, Sammons Preston, Rolyon, Bolingbrook, IL) by Southampton protocol
   10. Timed-Up-Go test
   11. 1 kg arm lift test – standard 1 kg dumbbell was used and participant asked to lift above head. If they were able to do, then time taken to do it repeat it for 5 times is noted in seconds.
   12. 5 times chair stand test
   13. Balance test: side by side stand, semi-tandem stand, tandem stand
   14. Functional reach test

**Follow up assessment:**

1. Any illness in the past year
2. Hospitalization in the past year
3. Indication for hospitalization
4. Fall in past year
5. Barthel ADL score
6. Overall self-rated health in past year: Excellent / Very good / Good / Fair / Poor
7. Death in past year

Table 1: Modified Kuppuswamy scale

| **Education of head of family** | **Score** | |
| --- | --- | --- |
| Profession or honours | 7 | |
| Graduate or postgraduate | 6 | |
| Intermediate or post high school diploma | 5 | |
| High school certificate | 4 | |
| Middle school certificate | 3 | |
| Primary school certificate | 2 | |
| Literate | 1 | |
| **Occupation of head of family** |  | |
| Profession | 10 | |
| Semi-profession | 6 | |
| Clerical, Shop-owner | 5 | |
| Skilled worker | 4 | |
| Semi-skilled worker | 3 | |
| Unskilled worker | 2 | |
| Unemployed | 1 | |
| **Monthly income of family** |  | |
| >41430 | 12 | |
| 20715-41429 | 10 | |
| 15536-20714 | 6 | |
| 10357-15535 | 4 | |
| 6214-10356 | 3 | |
| 2092-6213 | 2 | |
| <2091 | 1 | |
| **Socioeconomic class** | **Total score** | |
| I | Upper | 26-29 |
| II | Upper middle | 16-25 |
| III | Lower middle | 11-15 |
| IV | Upper lower | 5-10 |
| V | Lower | <5 |
